# Supplementary material for: Molecular Epidemiology of Respiratory Syncytial Virus during 2019–2022 and Surviving Genotypes after the COVID-19 Pandemic in Japan
Source: Viruses. 2023 Dec 4;15(12):2382. doi: 10.3390/v15122382 (PMC10748361; doi:10.3390/v15122382)
Supplement: Supplementary file 1 [file viruses-15-02382-s001.zip › viruses-2751738-supplementary.pdf]

Supplementary materials

Table S1. List of respiratory syncytial virus (RSV) glycoprotein (G) ectodomain region sequences used to create Maximum Likelihood (ML) and Bayesian Markov chain Monte Carlo (MCMC) trees.

Subgroup	Genotype	Location (prefecture)	Strain name	GISAID_ID
A	ON1	Aomori	RSA/Aomori/19RS46/2019_16-08-2019	EPI_ISL_16003290
A	ON1	Aomori	RSA/Aomori/19RS49/2019_29-08-2019	EPI_ISL_16003291
A	ON1	Aomori	RSA/Aomori/19RS50/2019_30-08-2019	EPI_ISL_16003292
A	ON1	Aomori	RSA/Aomori/19RS63/2019_13-09-2019	EPI_ISL_16003302
A	ON1	Hokkaido	RSA/Hokkaido/19RS112/2019_19-11-2019	EPI_ISL_16003320
A	ON1	Hokkaido	RSA/Hokkaido/19RS69/2019_10-09-2019	EPI_ISL_16003307
A	ON1	Hokkaido	RSA/Hokkaido/19RS72/2019_24-09-2019	EPI_ISL_16003309
A	ON1	Hokkaido	RSA/Hokkaido/20RS217/2021_05-07-2021	EPI_ISL_15980606
A	ON1	Hokkaido	RSA/Hokkaido/21RS28/2021_01-11-2021	EPI_ISL_15953497
A	ON1	Hokkaido	RSA/Hokkaido/21RS31/2021_11-11-2021	EPI_ISL_15953498
A	ON1	Hokkaido	RSA/Hokkaido/21RS36/2021_20-12-2021	EPI_ISL_15953499
A	ON1	Hokkaido	RSA/Hokkaido/21RS41/2022_01-02-2022	EPI_ISL_15953686
A	ON1	Hokkaido	RSA/Hokkaido/21RS42/2021_07-02-2022	EPI_ISL_15953948
A	ON1	Hokkaido	RSA/Hokkaido/22RS10/2022_13-09-2022	EPI_ISL_15953180
A	ON1	Kagawa	RSA/Kagawa/19RS20/2019_26-08-2019	EPI_ISL_16003280
A	ON1	Kagawa	RSA/Kagawa/19RS94/2019_07-10-2019	EPI_ISL_16003314
A	ON1	Kagawa	RSA/Kagawa/22RS11/2022_01-09-2022	EPI_ISL_15953181
A	ON1	Kagawa	RSA/Kagawa/22RS12/2022_05-09-2022	EPI_ISL_15953234
A	ON1	Kagawa	RSA/Kagawa/22RS16/2022_03-10-2022	EPI_ISL_15953393
A	ON1	Kagawa	RSA/Kagawa/22RS19/2022_24-10-2022	EPI_ISL_15953394
A	ON1	Kumamoto	RSA/Kumamoto/19RS106/2019_28-10-2019	EPI_ISL_16003317
A	ON1	Kumamoto	RSA/Kumamoto/19RS108/2019_16-11-2019	EPI_ISL_16003318
A	ON1	Kumamoto	RSA/Kumamoto/19RS31/2019_17-09-2019	EPI_ISL_16003283
A	ON1	Kumamoto	RSA/Kumamoto/19RS32/2019_17-09-2019	EPI_ISL_16003284
A	ON1	Kumamoto	RSA/Kumamoto/20RS11/2021_02-04-2021	EPI_ISL_15969898
A	ON1	Kumamoto	RSA/Kumamoto/22RS39/2022_24-11-2022	EPI_ISL_17280741
A	ON1	Nara	RSA/Nara/19RS192/2019_07-10-2019	EPI_ISL_16003333
A	ON1	Nara	RSA/Nara/19RS216/2019_09-12-2019	EPI_ISL_16003335
A	ON1	Nara	RSA/Nara/19RS221/2020_14-01-2020	EPI_ISL_16003337
A	ON1	Nara	RSA/Nara/20RS120/2021_27-05-2021	EPI_ISL_15970304
A	ON1	Nara	RSA/Nara/20RS125/2021_29-05-2021	EPI_ISL_15979224
A	ON1	Nara	RSA/Nara/20RS192/2021_12-07-2021	EPI_ISL_15979731
A	ON1	Nara	RSA/Nara/20RS193/2021_12-07-2021	EPI_ISL_15979732
A	ON1	Nara	RSA/Nara/20RS202/2021_14-07-2021	EPI_ISL_15980518
A	ON1	Nara	RSA/Nara/20RS204/2021_14-07-2021	EPI_ISL_15980520
A	ON1	Nara	RSA/Nara/20RS207/2021_15-07-2021	EPI_ISL_15980521

A	ON1	Nara	RSA/Nara/20RS215/2021_28-07-2021	EPI_ISL_15980605
A	ON1	Nara	RSA/Nara/20RS89/2021_30-04-2021	EPI_ISL_15969927
A	ON1	Nara	RSA/Nara/20RS96/2021_17-05-2021	EPI_ISL_15969931
A	ON1	Niigata	RSA/Niigata/19RS1/2019_20-08-2019	EPI_ISL_16003269
A	ON1	Niigata	RSA/Niigata/19RS103/2019_08-11-2019	EPI_ISL_16003316
A	ON1	Niigata	RSA/Niigata/19RS225/2020_31-01-2020	EPI_ISL_16003358
A	ON1	Niigata	RSA/Niigata/19RS6/2019_02-09-2019	EPI_ISL_18487960
A	ON1	Niigata	RSA/Niigata/19RS74/2019_03-10-2019	EPI_ISL_16003311
A	ON1	Okinawa	RSA/Okinawa/18U71/2019_23-07-2019	EPI_ISL_15980613
A	ON1	Okinawa	RSA/Okinawa/19U2/2019_06-08-2019	EPI_ISL_16003416
A	ON1	Okinawa	RSA/Okinawa/19U6/2019_02-08-2019	EPI_ISL_16003417
A	ON1	Okinawa	RSA/Okinawa/21U10/2021_06-09-2021	EPI_ISL_15954717
A	ON1	Okinawa	RSA/Okinawa/21U101/2021_22-11-2021	EPI_ISL_15967450
A	ON1	Okinawa	RSA/Okinawa/21U104/2021_29-11-2021	EPI_ISL_15967714
A	ON1	Okinawa	RSA/Okinawa/21U118/2021_14-12-2021	EPI_ISL_15969109
A	ON1	Okinawa	RSA/Okinawa/21U128/2021_27-12-2021	EPI_ISL_15969611
A	ON1	Okinawa	RSA/Okinawa/21U14/2021_14-09-2021	EPI_ISL_15969795
A	ON1	Okinawa	RSA/Okinawa/21U74/2021_26-10-2021	EPI_ISL_15967337
A	ON1	Okinawa	RSA/Okinawa/22U1/2022_02-09-2022	EPI_ISL_15953468
A	ON1	Okinawa	RSA/Okinawa/22U28/2022_19-10-2022	EPI_ISL_16842861
A	ON1	Okinawa	RSA/Okinawa/22U33/2022_26-10-2022	EPI_ISL_16844016
A	ON1	Okinawa	RSA/Okinawa/22U38/2022_04-11-2022	EPI_ISL_16844020
A	ON1	Okinawa	RSA/Okinawa/22U4/2022_10-09-2022	EPI_ISL_15953469
A	ON1	Okinawa	RSA/Okinawa/22U45/2022_22-11-2022	EPI_ISL_16844028
A	ON1	Okinawa	RSA/Okinawa/22U9/2022_20-09-2022	EPI_ISL_15953478
A	ON1	Shiga	RSA/Shiga/19RS115/2019_24-10-2019	EPI_ISL_16003321
A	ON1	Shiga	RSA/Shiga/19RS116/2019_28-10-2019	EPI_ISL_16003322
A	ON1	Shiga	RSA/Shiga/19RS120/2019_25-11-2019	EPI_ISL_16003324
A	ON1	Shiga	RSA/Shiga/19RS43/2019_13-09-2019	EPI_ISL_16003289
A	ON1	Shiga	RSA/Shiga/21RS61/2022_20-06-2022	EPI_ISL_15954709
A	ON1	Shiga	RSA/Shiga/21RS62/2022_22-06-2022	EPI_ISL_15954711
A	ON1	Shiga	RSA/Shiga/21RS65/2022_23-06-2022	EPI_ISL_15954715
A	ON1	Shiga	RSA/Shiga/22RS1/2022_10-09-2022	EPI_ISL_15953175
A	ON1	Shiga	RSA/Shiga/22RS21/2022_26-10-2022	EPI_ISL_16842706
A	ON1	Shiga	RSA/Shiga/22RS5/2022_17-09-2022	EPI_ISL_15953177
A	ON1	Tokyo	RSA/Tokyo/19RS102/2019_31-10-2019	EPI_ISL_16003315
A	ON1	Tokyo	RSA/Tokyo/19RS124/2019_10-12-2019	EPI_ISL_16003325
A	ON1	Tokyo	RSA/Tokyo/19RS125/2019_23-12-2019	EPI_ISL_16003326
A	ON1	Tokyo	RSA/Tokyo/19RS126/2019_27-12-2019	EPI_ISL_16003327
A	ON1	Tokyo	RSA/Tokyo/19RS15/2019_02-09-2019	EPI_ISL_16003278
A	ON1	Tokyo	RSA/Tokyo/19RS2/2019_08-08-2019	EPI_ISL_16003271
A	ON1	Tokyo	RSA/Tokyo/19RS3/2019_27-08-2019	EPI_ISL_16003272

A	ON1	Yamaguchi	RSA/Yamaguchi/19RS128/2019_09-09-2019	EPI_ISL_16003328
A	ON1	Yamaguchi	RSA/Yamaguchi/19RS132/2019_07-10-2019	EPI_ISL_16003331
A	ON1	Yamaguchi	RSA/Yamaguchi/19RS134/2019_24-10-2019	EPI_ISL_16003332
A	ON1	Yamaguchi	RSA/Yamaguchi/19RS230/2020_08-01-2020	EPI_ISL_16003359
A	ON1	Yamaguchi	RSA/Yamaguchi/19RS9/2019_21-08-2019	EPI_ISL_16003274
A	ON1	Yamaguchi	RSA/Yamaguchi/20RS236/2021_12-07-2021	EPI_ISL_15980608
A	ON1	Yamaguchi	RSA/Yamaguchi/20RS27/2021_06-05-2021	EPI_ISL_15969922
A	ON1	Yamaguchi	RSA/Yamaguchi/20RS36/2021_08-06-2021	EPI_ISL_15969924
A	ON1	Yamaguchi	RSA/Yamaguchi/21RS49/2021_25-11-2021	EPI_ISL_15954275
A	ON1	Yamaguchi	RSA/Yamaguchi/22RS24/2022_17-08-2022	EPI_ISL_16842849
A	ON1	Yamaguchi	RSA/Yamaguchi/22RS27/2022_09-09-2022	EPI_ISL_16842851
A	ON1	Yamaguchi	RSA/Yamaguchi/22RS35/2022_14-11-2022	EPI_ISL_16842856
A	ON1	Yamaguchi	RSA/Yamaguchi/22RS37/2022_02-12-2022	EPI_ISL_16842858
A	ON1	Yamaguchi	RSA/Yamaguchi/22RS41/2022_21-12-2022	EPI_ISL_17280742
B	BA9	Aomori	RSB/Aomori/19RS48/2019_29-08-2019	EPI_ISL_16013992
B	BA9	Aomori	RSB/Aomori/19RS61/2019_11-09-2019	EPI_ISL_16014366
B	BA9	Aomori	RSB/Aomori/19RS65/2019_02-10-2019	EPI_ISL_16014367
B	BA9	Hokkaido	RSB/Hokkaido/19RS71/2019_17-09-2019	EPI_ISL_16014443
B	BA9	Hokkaido	RSB/Hokkaido/21RS15/2021_16-09-2021	EPI_ISL_16003695
B	BA9	Hokkaido	RSB/Hokkaido/21RS17/2021_21-09-2021	EPI_ISL_16003697
B	BA9	Hokkaido	RSB/Hokkaido/21RS18/2021_04-10-2021	EPI_ISL_16003698
B	BA9	Hokkaido	RSB/Hokkaido/21RS22/2021_28-10-2021	EPI_ISL_16003702
B	BA9	Hokkaido	RSB/Hokkaido/21RS33/2021_26-11-2021	EPI_ISL_16003706
B	BA9	Kagawa	RSB/Kagawa/19RS21/2019_05-09-2019	EPI_ISL_16013984
B	BA9	Kagawa	RSB/Kagawa/19RS93/2019_30-09-2019	EPI_ISL_16014446
B	BA9	Kagawa	RSB/Kagawa/19RS98/2019_28-10-2019	EPI_ISL_16014528
B	BA9	Kagawa	RSB/Kagawa/22RS14/2022_15-09-2022	EPI_ISL_16003687
B	BA9	Kagawa	RSB/Kagawa/22RS15/2022_20-09-2022	EPI_ISL_16003688
B	BA9	Kumamoto	RSB/Kumamoto/19RS33/2019_17-09-2019	EPI_ISL_16013988
B	BA9	Kumamoto	RSB/Kumamoto/20RS16/2021_15-05-2021	EPI_ISL_16003802
B	BA9	Kumamoto	RSB/Kumamoto/21RS27/2021_03-11-2021	EPI_ISL_16003704
B	BA9	Kumamoto	RSB/Kumamoto/21RS37/2021_20-12-2021	EPI_ISL_16003707
B	BA9	Kumamoto	RSB/Kumamoto/21RS38/2022_17-01-2022	EPI_ISL_16003708
B	BA9	Kumamoto	RSB/Kumamoto/21RS77_2022_22-02-2022	EPI_ISL_18487961
B	BA9	Mie	RSB/Mie/20RS221/2021_01-04-2021	EPI_ISL_16003904
B	BA9	Mie	RSB/Mie/20RS223/2021_26-04-2021	EPI_ISL_16003905
B	BA9	Mie	RSB/Mie/20RS231/2021_16-06-2021	EPI_ISL_16004072
B	BA9	Nara	RSB/Nara/19RS142/2019_10-08-2019	EPI_ISL_16014533
B	BA9	Nara	RSB/Nara/19RS238/2020_20-01-2020	EPI_ISL_16014542
B	BA9	Nara	RSB/Nara/19RS244/2020_19-02-2020	EPI_ISL_16014576
B	BA9	Nara	RSB/Nara/20RS127/2021_31-05-2021	EPI_ISL_16003889
B	BA9	Nara	RSB/Nara/20RS177/2021_29-06-2021	EPI_ISL_16003895

B	BA9	Nara	RSB/Nara/20RS211/2021_24-07-2021	EPI_ISL_16003898
B	BA9	Niigata	RSB/Niigata/19RS35/2019_24-09-2019	EPI_ISL_16013989
B	BA9	Niigata	RSB/Niigata/19RS36/2019_24-09-2019	EPI_ISL_16013991
B	BA9	Okinawa	RSB/Okinawa/18U48/2019_04-07-2019	EPI_ISL_16013981
B	BA9	Okinawa	RSB/Okinawa/19U21/2019_20-09-2019	EPI_ISL_16014578
B	BA9	Okinawa	RSB/Okinawa/20U20/2020_22-10-2020	EPI_ISL_16013974
B	BA9	Okinawa	RSB/Okinawa/20U82/2020_11-12-2020	EPI_ISL_16013978
B	BA9	Okinawa	RSB/Okinawa/21U4/2021_31-08-2021	EPI_ISL_16003721
B	BA9	Okinawa	RSB/Okinawa/21U78/2021_26-10-2021	EPI_ISL_16003718
B	BA9	Okinawa	RSB/Okinawa/21U8/2021_04-09-2021	EPI_ISL_16003725
B	BA9	Okinawa	RSB/Okinawa/22U30/2022_21-10-2022	EPI_ISL_16844030
B	BA9	Okinawa	RSB/Okinawa/22U41/2022_16-11-2022	EPI_ISL_16844031
B	BA9	Shiga	RSB/Shiga/20RS10/2021_06-05-2021	EPI_ISL_16003800
B	BA9	Shiga	RSB/Shiga/20RS7/2021_26-04-2021	EPI_ISL_16003799
B	BA9	Tokyo	RSB/Tokyo/19RS79/2019_03-10-2019	EPI_ISL_16014444
B	BA9	Tokyo	RSB/Tokyo/20RS46/2021_03-07-2021	EPI_ISL_16003832
B	BA9	Tokyo	RSB/Tokyo/20RS51/2021_19-07-2021	EPI_ISL_16003880
B	BA9	Tokyo	RSB/Tokyo/20RS54/2021_29-07-2021	EPI_ISL_16003887
B	BA9	Tokyo	RSB/Tokyo/21RS1/2021_02-08-2021	EPI_ISL_16003692
B	BA9	Tokyo	RSB/Tokyo/21RS3/2021_10-08-2021	EPI_ISL_16003693
B	BA9	Yamaguchi	RSB/Yamaguchi/19RS10/2019_26-08-2019	EPI_ISL_16013983
B	BA9	Yamaguchi	RSB/Yamaguchi/20RS25/2021_28-04-2021	EPI_ISL_16003828
B	BA9	Yamaguchi	RSB/Yamaguchi/20RS31/2021_17-05-2021	EPI_ISL_16003830
B	BA9	Yamaguchi	RSB/Yamaguchi/21RS48/2021_22-11-2021	EPI_ISL_16003709
B	BA9	Yamaguchi	RSB/Yamaguchi/21RS54/2021_25-12-2021	EPI_ISL_16003713
B	BA9	Yamaguchi	RSB/Yamaguchi/22RS31/2022_03-10-2022	EPI_ISL_16844029

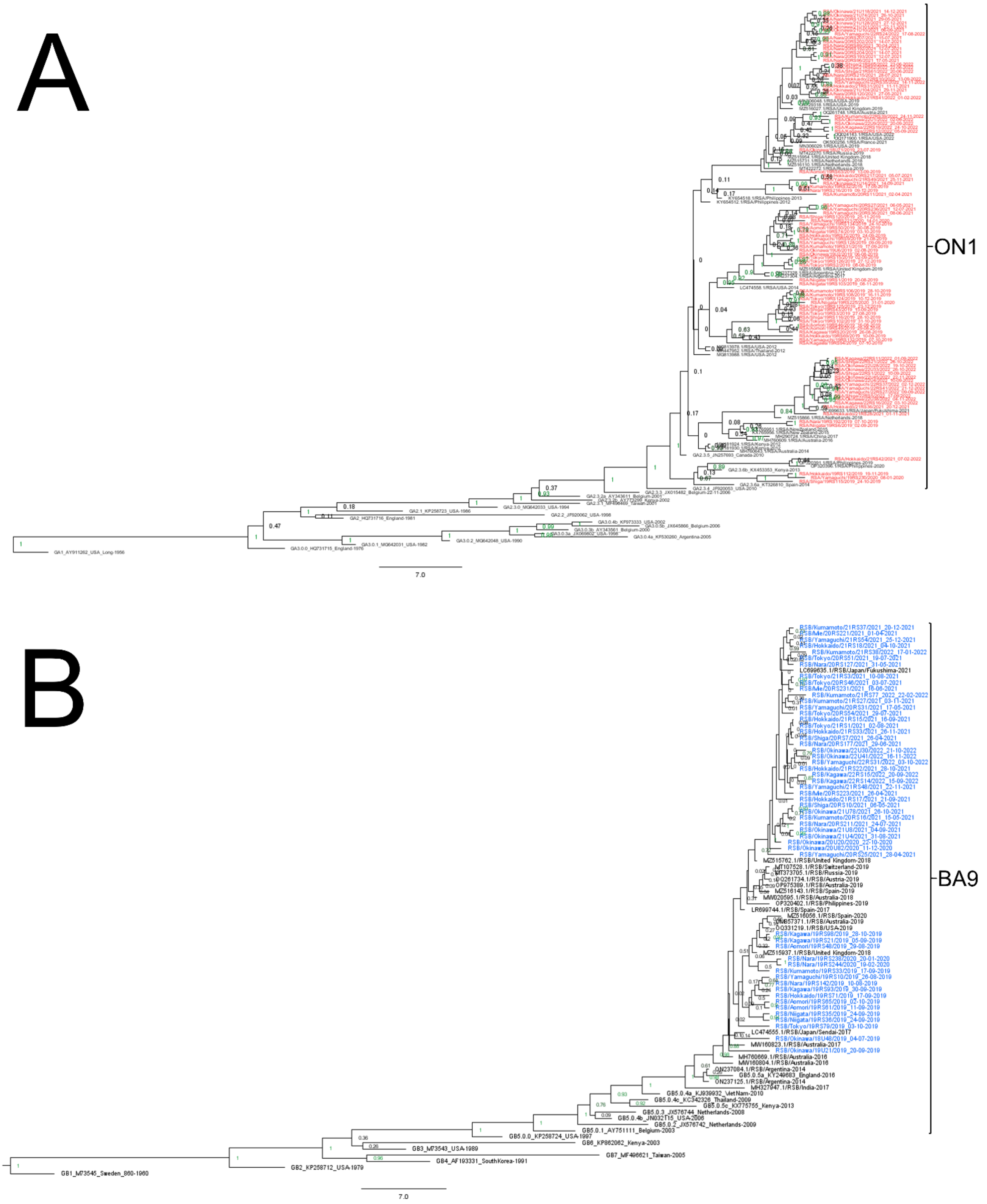

Figure S1. Phylogenetic tree analysis of respiratory syncytial virus (RSV) strains circulating in Japan from 2019 to 2022. Bayesian inference phylogenetic trees of glycoprotein (G) ectodomain region gene of RSV-A (A) and RSV-B (B) were generated by BEAST 1.10.4 software. RSV-A strains obtained in this study are colored red, RSV-B strains are colored blue and reference strains from Japan and the other countries were colored black. The sequences obtained in this study were named using the RSA/B-collected prefecture-number of specimen-collection "year_ collection date format. Only posterior ≥ 0.8 is colored green at the branch nodes. The genotypes are shown on the right square brackets.
